# Transition to online psychological support – Barriers, stereotypes and challenges from the perspective of service providers

**DOI:** 10.1111/papt.70010

**Published:** 2025-08-27

**Authors:** Miroslav Charvát, Leona Jochmannová, Petra Zia Sluková, Lucie Viktorová

**Affiliations:** ^1^ Department of Psychology Faculty of Arts, Palacký University Olomouc Olomouc Czechia

**Keywords:** barriers, counselling, embodiment, online psychological care, psychotherapy, reflexive thematic analyses, telehealth

## Abstract

**Objectives:**

This qualitative study, which provides practical insights for the field, described the transition process of psychological service providers towards online care provision. We aimed to describe the process of professionals' adaptation to new methods of distant psychological care, including its main facilitating and complicating elements.

**Methods:**

We used a qualitative approach, specifically evaluation design, employing reflexive thematic analyses conducted by the software ATLAS.ti. Semi‐structured interviews were conducted with 51 professionals, and 14 focus groups with 44 experts. We worked with 95 experienced professionals specializing in clinical psychology, psychological counseling, and counseling those affected by domestic violence. All of them have had practical exposure to delivering care online.

**Results:**

The results show that after first‐hand involvement, most of the providers have moved from being cautious experimenters to being regular practitioners of online remote psychological care in cases where it is appropriate. Technical difficulties and negative stereotypes receded, with many respondents striving to provide online services for their clients' benefit. However, some negative aspects persist, including the lack of timely support from key institutions.

**Conclusions:**

Logistical barriers stem from poor management and insufficient political, legislative and financial support. Ethical and legal challenges require serious attention from key system players. The question of embodiment, involving new therapeutic cues and compensating for absent ones, warrants thorough follow‐up research. Bias and stereotype‐related barriers, often shaped by providers' attitudes, can be addressed through targeted training and shared professional experiences.

## INTRODUCTION

The transition to online psychological care represents a significant shift in the delivery of mental health services, raising essential questions about the adaptability of service providers, the challenges they face, and the stereotypes that influence their attitudes. Despite the growing prevalence of telehealth modalities, existing research has primarily focused on clients' perspectives or technological feasibility, leaving a gap in understanding the experiences and beliefs of psychological professionals tasked with implementing these approaches. This study explores the barriers, facilitators, and evolving attitudes of psychological service providers, particularly clinical psychologists and psychological counsellors. The present study examines the most frequent challenges, barriers, and supportive factors for implementing and delivering videoconferencing in everyday practice.

In this paper, ‘telemental health’ or ‘distant care’ primarily refers to interactive videoconferencing exchanging real‐time visual and auditory stimuli (Nelson & Velasquez, 2011), where the medium is associated with reasonable user satisfaction and outcomes similar to in‐person therapy delivery (Backhaus et al., 2012; Varker et al., 2019). The concept also includes various tools and other platforms that create organisational support for videoconferencing, e.g., asynchronous (email) and synchronous (instant messaging) communication modalities. Many definitions apply to similar constructs; they are often used interchangeably and mainly refer to the usage of information and communication technologies (ICT): online therapy, distance therapy, e‐therapy, e‐counselling, e‐psychology, telepsychology, teletherapy, online psychotherapy, online counselling, digital therapy, internet or web therapy, computer‐assisted therapy, etc. (Perle, 2021; Stoll et al., 2020; Tuerk & Shore, 2015).

Many psychological care providers have had the opportunity or been required to adapt to provide psychological care online in recent years, for example, during the COVID‐19 pandemic, but also in response to emerging client needs or preferences. However, most of them were not familiar with videoconferencing (Machluf et al., 2021; Mendes‐Santos et al., 2020) or had a slightly pessimistic attitude towards it (Berger, 2017; Centore & Milacci, 2008; Hines et al., 2015). The COVID‐19 lockdowns caused changes in attitudes towards the online modality. Providers reported high levels of satisfaction, acceptability, and appropriateness in connection with the increased use of videoconferencing (Doran & Lawson, 2021; Gentry et al., 2021; Li et al., 2021; Machluf et al., 2021).

The most frequently cited barriers while implementing telemental health modalities included technical issues (Bierbooms et al., 2020; Connolly et al., 2020; Dijksman et al., 2017); lack of technological literacy (Kotera et al., 2021; Schuster et al., 2018; Terry & Buntoro, 2021) or perceived difficulty of use (Gilmore & Ward‐Ciesielski, 2019; McKee et al., 2021); privacy, confidentiality, and lack of intimacy (Austen & McGrath, 2006; Fletcher et al., 2018); the difficulty of connecting nonverbally or reacting appropriately to clients' emotions (Bierbooms et al., 2020; Kotera et al., 2021; Schuster et al., 2018); financial and legal issues including payments, insurance, and reimbursement policies (Lin et al., 2018; Mol et al., 2019), and administrative and logistical challenges, also covering organisational processes (Muir et al., 2020; Myers et al., 2020).

One of the main concerns is a lack of specific knowledge and professional training to ensure successful online interventions (Dijksman et al., 2017; Gentry et al., 2021; Hanley, 2006; Mendes‐Santos et al., 2020; Tohme et al., 2021). The need for specific manuals (Jochmannová et al., 2023) or training programs aimed at increasing providers' knowledge and competence, such as the Telemental Health (TMH) training programme (Felker et al., 2021), has also increased.

The positive beliefs about the modality were associated with perceptions of its consistency with traditional therapy in terms of effectiveness, strong therapeutic alliance, and a perceived sense of safety, rated by both clients and providers (Andersson et al., 2014; Barnett et al., 2021; Hilty et al., 2013; Hines et al., 2015; Jenkins‐Guarnieri et al., 2015; Martin et al., 2000). Providers' concerns (Cowan et al., 2019; Muir et al., 2020), a sense of control (Smith & Gillon, 2021), acceptance and the ability to adapt to new technology (Brooks et al., 2013; Wagnild et al., 2006), and resistance to change (Scott et al., 2018) represent other essential factors in the adoption of telemental health.

Some findings suggest that external factors such as the necessity of care (Gentry et al., 2021), usefulness in the context of improved access for clients (Gilmore & Ward‐Ciesielski, 2019; Yoon et al., 2021), having dedicated staff responsible for promoting and managing the new service, or organisational policies supporting telemental health modalities (Muir et al., 2020; Pierce et al., 2021) also influence the implementation of online services.

The most common barriers reported were clinician‐related, specifically clinicians' attitudes (Cowan et al., 2019; Muir et al., 2020; Springer et al., 2020). According to Wagnild et al. (2006), acceptance by clinicians is a central principle in the expansion and sustainability of telemental health, more so than technological problems or a lack of resources. Openness to new treatments and positive attitudes are key to implementing new technologies (Schuster et al., 2020) and are often associated with therapists' previous experience with the online modality (Austen & McGrath, 2006; Connolly et al., 2020; Doran & Lawson, 2021; Interian et al., 2018; Tohme et al., 2021) or professional experience (Cwikel & Enav, 2020; McLeod, 2007; Schuster et al., 2020). However, Muir et al. (2020) reported that less rational anxieties, such as concern over one's appearance or a fear of appearing foolish, may not be so easily alleviated. Most of the providers indicated that their concerns and negative beliefs tended to fade after engaging with the online modality during COVID‐19 (Machluf et al., 2021).

Online psychotherapy is popular and effective, but its capacity to support the implicit nonverbal and embodied aspects of the therapeutic relationship has been questioned and remains understudied. The conceptual framework integrates embodiment as the lived bodily experience influencing therapeutic interaction (García et al., 2022) and nonverbal communication as the transmission of affective and relational cues (Lin & Anderson, 2024). It is the therapeutic relationship as a dynamic alliance between therapist and client. These concepts are interrelated, with embodiment underpinning nonverbal synchrony and presence, which in turn shape the quality and efficacy of the therapeutic relationship (Jennissen et al., 2024). This framework guides the exploration of how online modalities modify these processes and inform clinician adaptation.

The study aimed to describe the process of professionals' adaptation to new methods of distant psychological care, including its main facilitating and complicating elements caused by the absence of face‐to‐face contact with their clients.

## METHODS

We used a qualitative approach, specifically evaluation design, employing Reflexive thematic analyses (Braun & Clarke, 2022). Data were collected using semi‐structured interviews and focus groups, held and recorded using online applications (Zoom, Skype, and MS Teams). The questions targeted the following areas of interest: reflection on experience with distant psychological care, significant challenges and issues concerning types of interventions, identification of suitable and unsuitable clients, technical aspects of distant care, and recommendations for future practice. The individual interviews generally lasted between 40 and 60 min, whereas the focus group sessions ranged from 90 to 120 min. The interviews and focus groups were conducted, transcribed, and analysed in Czech. Only quotations used in this paper were translated into English by a professional translator for the purposes of publication, ensuring that their meaning and authenticity were preserved. A body of verbatim transcripts that amounted to approximately 1,064,000 characters without spaces, or 232,000 words, was generated. In accordance with research ethics and the applicable EU regulations (such as the GDPR), all identifying information related to specific individuals and workplaces was removed from the text. This anonymised corpus was analysed by coding and grouping it into thematic units using ATLAS.ti Version 9 (Friese, 2019). The body of transcripts of such a size resulted from the effort to achieve data saturation. To enhance the validity of the results, we relied on procedures involving data source triangulation, triangulation of data collection methods, and the triangulation of the perspectives of multiple psychological disciplines.

## PARTICIPANTS

We used purposive, criterion‐based sampling to capture a broad range of psychologists experienced in remote service provision in Czechia. We circulated an invitation email (with an attached participant‐information sheet) through the national professional bodies for psychology (e.g. the Association of Clinical Psychologists of the Czech Republic) and through five cooperating institutions that had served as practice guarantors in our grant application. In addition, subject matter experts known for their publications or training activities in telepsychology were contacted individually.

We worked with 95 participants (three‐quarters were women, a typical proportion for psychology). The age range was from 30 to 57 years. All met the inclusion criteria; specifically, they were experienced psychologists (with a minimum of five years of professional practice) who provide psychological and therapeutic services to clients of various ages, and their daily practice mainly includes interventions related to psychotherapy, counselling, and psychological assessment. The participating psychologists were members of professional organisations and had completed long‐term psychotherapeutic training. Additionally, participants were required to have experience in delivering care online. It was not formally quantified; however, all had provided such services for at least two years across successive waves of the COVID‐19 pandemic. Caseloads varied markedly from the low tens to the low hundreds of clients, particularly in crisis intervention and diagnostic work. We conducted 51 individual interviews, 16 involving clinical psychologists and 35 psychologists outside the clinical setting (i.e., practitioners providing psychotherapy or other psychological services without ties to the public health insurance system, either through direct client payments in private practice or within the non‐profit sector). Additionally, we organised 14 focus groups covering a more focused and in‐depth discussion of selected subtopics (issues of psychotherapy, psychological assessment, and online counselling) with another 44 psychologists (3–5 participants per group). In the Czech Republic, working in the healthcare system requires accredited postgraduate specialised training lasting five years, concluded with a board certification examination to qualify as a clinical psychologist. Outside the healthcare system, practising as a psychotherapist requires a master's‐level university degree and comprehensive long‐term psychotherapeutic training.

Participants received both written and oral information prior to each interview or focus group session, explaining that the study aimed to identify the main challenges of distant psychological care and to develop evidence‐based online protocols for psychodiagnostics, psychotherapy, and counselling in situations where direct, face‐to‐face care is not feasible. They were informed that participation was voluntary, non‐remunerated, and could be terminated at any time; sessions would be video‐recorded and subsequently anonymised; all data would remain confidential within the research team. Participants were also asked to respect the confidentiality of others' contributions. Furthermore, they were informed of their right to request data erasure prior to anonymisation and to obtain the study's results. All of them signed a written informed consent to participate in the research and allowed the use of the collected data for research purposes.

The research participants were professional providers of psychological services with self‐experience training; they were questioned solely about the content and processes of their professional work, not about sensitive personal data. Moreover, all interviews were conducted sensitively by experienced psychologists, so the possibility of harm was considerably lower than it would be for client participants.

Funding was obtained from the Technology Agency of the Czech Republic, a state organisational unit founded to support research, experimental development, and innovation. The complete project proposal, including methodology and ethical considerations, was reviewed and approved by independent experts appointed by this national funding agency, and no ethical issues were identified. The study and its ethical and professional parameters were further endorsed by the practice guarantors: the Association of Clinical Psychologists of the Czech Republic, the Association of Intervention Centre Providers, the National Telemedicine Centre, and the Czech Association for Psychotherapy. They agreed and provided written consent.

## RESULTS

### General framework

**FIGURE 1:**
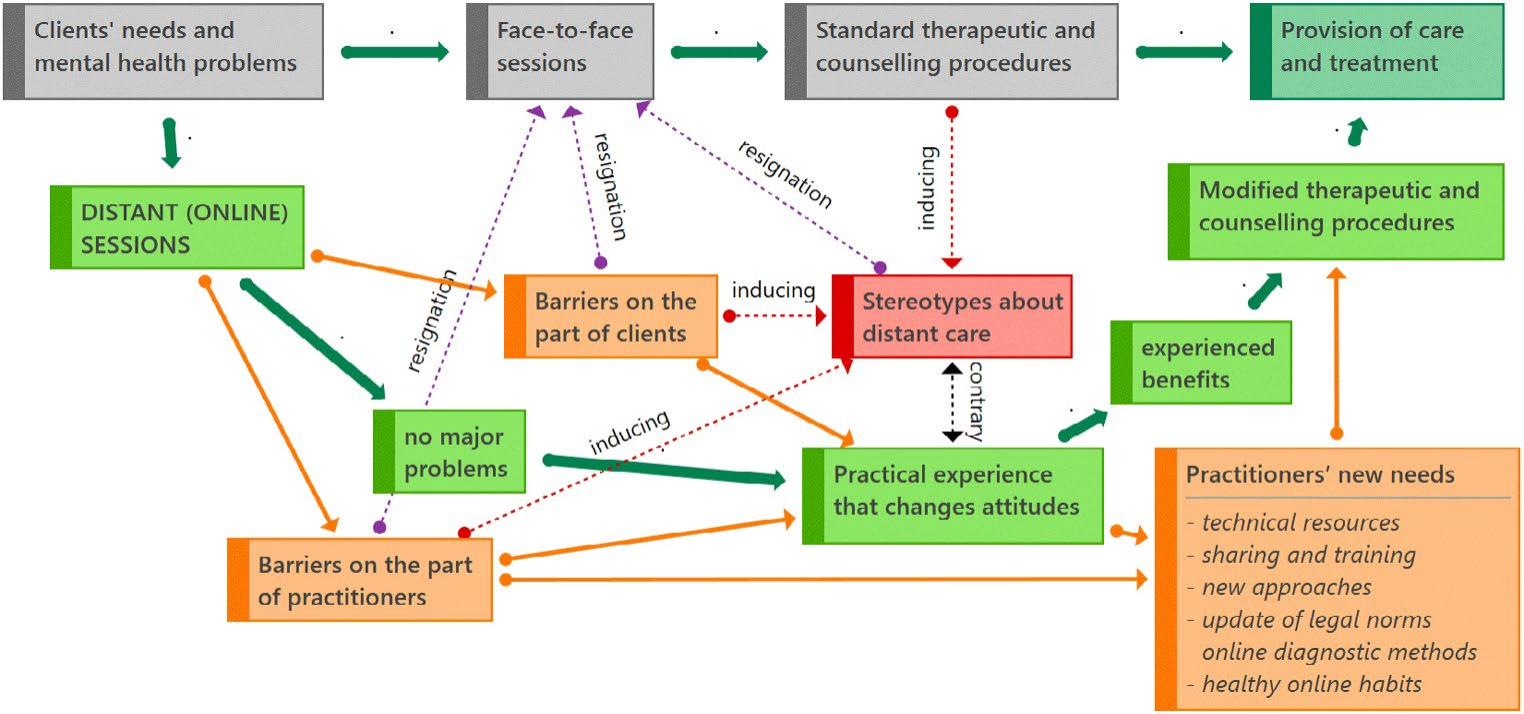
Process of transition to distant (online) psychological care and its barriers.

Before proceeding with thematic classification, we outlined a general framework (Figure 1). This diagram was developed inductively during the inductive theme development stage of our reflexive thematic analysis.

The provision of traditional psychological care is straightforward. Clients contact a psychologist or organisation with their difficulties. They meet face‐to‐face at an agreed time, arrange a contract, and the provision of counselling or therapy according to standard procedures can begin (process at the top of the diagram). However, a shift to online videoconferencing does not always proceed smoothly. Nevertheless, some professionals we interviewed reported surprisingly smooth transitions, accompanied by a growing appreciation of the benefits of distance care and a willingness to adapt standard psychotherapeutic procedures when necessary. In most of the cases, we noted several barriers and challenges on the part of both clients and providers that, at best, did not lead to the discontinuation of these but instead to the reflection of new needs and the implementation of necessary changes and modifications. Difficult obstacles, however, can also directly lead to resignation in providing psychological care online or induce or reinforce various stereotypes regarding the insufficiency of distance care and its qualitative incomparability with traditional face‐to‐face practices. However, complex barriers can also lead directly to resignation in the provision of online mental health care. They can also create or reinforce various stereotypes about the inadequacy of distance care and its qualitative incomparability to traditional face‐to‐face practice. However, on closer inspection and with increasing experience, these stereotypes are difficult to sustain unless providers are discouraged. However, there are objective barriers to some ways of working. The following is a discussion of the specific themes that have emerged.

The following is a summary of the main themes found and sub‐phenomena emerging from the Reflective Thematic Analysis. A summary can be found in Table 1.

**TABLE 1:** A summary of the main themes and phenomena.

Main themes	Problems/phenomena
Theme 1: Adaptational, logistic and technical barriers	Limited or no experience with videoconferencing and online care Inadequate technological resources for both clients and practitioners Challenges in adapting workflows to digital environments Technological glitches disrupting service delivery Lack of mental health practices and privacy challenges for both practitioners and clients Limited access to online psychological assessment tools and a lack of awareness among staff
Theme 2: Barriers concerning therapeutic contact and embodiment	Difficulty in establishing emotional intimacy and rapport online Loss of non‐verbal and para‐verbal cues affecting communication quality Limited ability to assess nonverbal signs remotely Fatigue associated with prolonged video interactions Clients' concerns over the authenticity of the therapeutic connection Challenges in handling crises effectively during remote sessions Professionals divide into three groups: those seeing distant care as an emergency measure only, a complementary alternative, or a progressive step forward
Theme 3: Ethical and legal barriers	Concerns over data security and client confidentiality Ambiguities in legal regulations for online therapy Ethical dilemmas related to digital consent and record‐keeping Unequal distribution of privacy risks across client populations Challenges in ensuring compliance with evolving legal frameworks Limited or no reimbursement for online interventions by health insurers reduced providers' incentives, leading to ethical dilemmas
Theme 4: Stereotypes versus experience	Resistance to online care due to preconceived notions about efficacy Underestimation of clients' willingness to adapt to digital formats Overgeneralization of initial negative experiences Initial scepticism about online care shifted with experience Certain client groups (e.g., those with severe mental disorders) were deemed unsuitable for online care

### Adaptational, technical and logistic barriers

The theme of adaptation primarily concerns the unplanned transition to online care delivery that participants experienced, particularly during the COVID‐19 pandemic, which was one of the first opportunities for many providers to experience working via videoconferencing. The psychosocial care sector was caught off guard by the unexpected restrictive measures to contain the pandemic. The first thoughts of many practitioners turned to possible ways of maintaining contact with clients or performing urgent procedures. Practitioners experienced a great deal of uncertainty.

In the case of unplanned intervention, the first choice for those who opted for a remote form of working with clients was the classic telephone contact, which is already an established standard approach for crisis intervention. When online video calls were implemented, the first limitations related to the lack of training and experience with the technical details of online interaction appeared. Especially among older practitioners, video conferencing was not a common way of communication until then. They were unfamiliar with the different types of applications preferred by clients, did not know how to set up their cameras and microphones properly, and used personal profiles rather than professional ones. Practitioners and their clients were only getting acquainted with such procedures, which sometimes took up dedicated psychological care time. Advice or training from technically savvy colleagues or IT specialists was useful for them, but not always available.

The general lack of information seemed closely related to the need to share with colleagues. At best, the employer holds intervention meetings with staff, especially in facilities with small staff. Larger organisations do not manage this very well. Their staff often had to rely on self‐help meetings arranged ad hoc or individual conversations with like‐minded colleagues.

The disadvantages and difficulties associated with poor mental health resulted from the increased time spent during the period of restrictive measures, especially working from home, and the increased number of hours spent in front of computer screens. Online contact fatigue developed over time. It is also difficult to ensure enough peace and privacy for working online. Another problem reported was setting limits on the availability of psychologists. Online contact is easier from home or outside working hours. ‘Then it is also important to find your fixed workplace, work at specific times from a specific place, and find a quiet setting. Do not forget about yourself and your mental health. It sounds like a cliché, but I feel it is important’. *(psychologist, 45 years old)*. This is closely related to the issue of privacy of clients, which is key to counselling and psychotherapeutic processes. Specifically, this issue was often about working with children, where their parents could have been covertly present in the same room. However, it generally referred to the undesirable presence of other people who may have somewhat eavesdropped the ongoing session.

Several logistical obstacles were due to insufficient crisis management on the part of the providers and the organisations they work for and inflexible support from the government. ‘I mean, just red tape… You need to have an online therapy session right away, or you need a phone to put a SIM card in. Such a trivial matter. But it's a public institution, so get ready for a bidding procedure and approval process, and before they buy you a telephone and a licence, the pandemic is over. So it came down to me buying the phone with my own money, and we bought the software licence with our own money, too’. *(focus group – clinical psychology)*. Some managers were not able to resume the system of adequate care and were left paralysed by the pandemic situation, while others minimised and downplayed the pandemic.

In many cases, the technical equipment that was available proved to be inadequate. There was a lack of agency telephones, notebooks, cameras, and software that met the desired technical criteria. The practitioners generally dealt with this by using their own personal devices. In many cases, those did not come close to the required configuration and performance. This was also complicated by the market situation, where the supply of optimal devices was limited at the time. This was even more apparent on the clients' part.

Usually sufficient to meet the demands of the household under normal circumstances, the internet connection was strained by children's online education and spouses working from home during pandemic restrictions, which led to the frequent slowing down of the data flow or failures. Large organisations such as hospitals had the advantage of IT personnel being available to help with technical procedures (such as opening accounts, software installation, and securing communication). On the other hand, large organisations were more prone to massive hacker attacks, which was the case with one healthcare facility.

These shortcomings were felt particularly keenly in the use of online psychological assessment. There were significant shortages of these resources in many organisations. The staff did not possess licences for using online assessment systems and were often unaware of the online assessment tools and approaches available on the market.

### Barriers concerning therapeutic contact and embodiment aspects

Once professionals started providing care online, they soon felt the need to obtain information that would help modify distant care approaches in such a way as to preserve the professional standards of the services. The need for new methodological protocols and specialist training was growing at the first stage. While the ministries launched several project calls intended to help this effect, the development of guidelines that are to be based on research evidence takes time. The support from professional associations or the key professional authorities and the bearers of know‐how in psychology is still somewhat sporadic. It does not meet the real needs of practice. The overall lack of knowledge was reflected again either in giving up on the possibility of delivering full‐fledged care or trying new approaches on a self‐help basis. Managers played a significant role in this process, in both a negative and positive sense: ‘The therapists just couldn't imagine how this could possibly be done. Some argued about the lack of evidence. And then our boss dug out some research from 2015 or so, and that, it seemed to me, got my colleagues convinced. So, it was really about some data they could build on’. *(Focus group – clinical psychology)*.

With online contact, it is difficult for some practitioners to ensure that concentration remains focused on the ongoing counselling or therapeutic process. A computer connected to the internet or the home environment can be highly distracting and tempt people to engage in other appealing activities or undesirable hidden parallel communication in another app. Clients were often more likely to forget about online appointments or cancel them. These problems were more pronounced among involuntary clients or clients from socially disadvantaged settings (clients exposed to domestic violence, children from dysfunctional families, clients of drug services, etc.). Many online ordering systems can address this, but clients are still learning to use them effectively.

The practitioners complained about the absence of face‐to‐face contact, as they could not watch clients' reactions, such as physical responses, body‐posture and interpersonal‐distance cues, and emotional expressions. Videoconferencing did not always provide enough details of the face, let alone make it possible to watch the client's entire body. From the majority's perspective, these embodied aspects are crucial for building rapport, empathy, and attunement between therapist and client. Paraverbal expressions (such as a quiver in the voice, raising of the voice, slips of the tongue) were more challenging to recognise, mainly because of the distorted sound transmitted by low‐quality microphones or, paradoxically, because of their capability of filtering out ambient noise and din. ‘It was mostly the moments when I missed the direct contact – pauses, silence, to read how the client responds from the expression on his face. It makes you uncertain and want to comment on things more. Instinctively, like automatically, I tended to do more talking. I was more in charge of the session. It then changes the framework towards more directive work’. *(clinical psychologist, 32 years old)*. This problem was brought up with even greater intensity about activities involving more clients at a time (pairs, families, groups), where, in addition, therapists could not monitor clients' responses to each other, and it was also more difficult to work with group dynamics or silence. Paradoxically, some clients found the less intensive contact more comfortable, primarily clients with anxiety with face‐to‐face contact, for whom online therapy may be more of a contraindication.

Psychologists at educational‐psychological counselling centres and clinical child psychologists complained specifically about the difficulty of performing psychodiagnostic procedures or specific therapeutic techniques, which typically require body work or the use of specific material. ‘We skipped online remedial interventions with boys with learning disorders. You can't see how he's holding the pencil, and when you tilt the camera, you can't see him for a change. That would require a system with multiple cameras. And there's no possibility of making immediate adjustments, just touching the hand or the arm’. *(Focus group – psychological assessment)*. An issue that was frequently raised in addiction counselling referred to the ways of providing interventions requiring contact or a supply of some specific material (e.g. mandatory testing for abstinence among clients of drug services). In this case, direct contact is a must.

### Ethical and legal barriers

Many professionals grew dissatisfied with continuing to engage in improvised and legally and ethically unsound procedures. Online contact via various videoconferencing apps raised concerns about the client's privacy. ‘So we were developing all kinds of guidelines and rules as to how all this should be done. And we chose a specific platform because we really made a point of addressing the issue of anonymity, which is crucial to us because otherwise, clients with sexual paraphilia won't engage’ *(focus group – clinical psychology)*. However, few understood feasible ways of securing their communication and data sent over the internet. So, they primarily relied on the reputation of their software or common security elements protecting their computers only. ‘I asked myself how a conversation with a patient could be misused really, as it is actually of no use, perhaps to a family member, but not to a hacker. Who would he be selling that to? I understand that when somebody hacks a hospital, then there the data can be sold, but who would be interested in buying a therapeutic conversation?’ *(focus group – clinical psychology)*. Similar concerns were raised about the sharing of computerised documents containing sensitive data. According to the practitioners, these transactions should involve using a specific type of informed consent, which had not been adapted for distant practice; the practitioners were unsure about the form and wording of such a document.

There was great uncertainty as to what could be formally performed as part of distant psychological care, what could be regarded as lege artis, and what could be contested on either professional or legal grounds. In an effort to help their clients, some bolder providers and organisations pursued distant psychological care without solid legal grounds, thus often finding themselves in a difficult situation. However, legislative changes take years to implement. These concerns grew in cases where a psychological assessment report was required to inform conclusions or decisions of critical importance for the life of a client (assessment for a disability allowance, differential psychodiagnosis, deferment of school attendance, results of court‐ordered treatment, reports for child protection services, etc.). Here again, we seem to be encountering objective limits to online psychological care.

Another important issue concerned the security of diagnostic tools and psychological tests. Sometimes the practitioners dealt with the shortage of the versions of the methods officially distributed for online use by sending scans of the methods in PDF format via email or converting them to online forms on a self‐help basis. Although they knew it was not a standard procedure and they might compromise the protection of the methods, it was often seen as the only way for them in a pandemic.

In mental health care, as far as clinical psychologists were concerned, there was a failure on the part of health insurers, i.e. state‐controlled institutions, as they did not reimburse online interventions or only did so to a limited degree compared to face‐to‐face services in a pandemic. For many professionals, this posed a great barrier and lowered their motivation to continue providing care. Work without full remuneration would be economically unsustainable for them in the long run. Some practitioners even admitted to reporting online care as if it had been performed face‐to‐face (or reported another intervention instead), as it was carried out simultaneously and at a comparable therapeutic quality. Nevertheless, they were annoyed by being indirectly pushed by the insurers to engage in such practices.

### Stereotypes or objective barriers?

The practitioners unnecessarily created some of the barriers by themselves. They seemed embedded in their attitudes and biases rather than insurmountable logistical or professional problems. ‘As for the clients, it never worked like that before in practice, to meet clients like this at a distance through video calls. Consultations over the phone, yes, we've had that. I don't think there's much that would prevent that from being put in place. Maybe there are some stereotypes that one may cling to, that's what it's probably about’ *(psychologist, 49 years old)*.

The most common techniques and activities reported as impossible to perform included online assessment and psychodiagnostics. ‘For one thing, assessment using diagnostic methods is hardly possible at all. I might try a Rorschach test, but to a very limited degree. I can't really imagine that’. *(clinical psychologist, 45 years old)*. Interestingly, for example, the work and organisational psychology sector has almost no problems with online assessments because it is equipped with and has widely used psychodiagnostic tools and licenses for online use.

There were also frequent reservations about pair, family, and group therapies, even where a practitioner had already had experience with distant individual care. ‘We went online with what we did previously individually, except for where this was not possible. … I have my reservations about that’. *(focus group – counselling)*.

The participants reported various types of clients whom they found utterly unsuitable for long‐distance interaction. Such clients included patients with decompensated mental disorders, particularly those involving paranoid states, mania, severe forms of depression, suicidal tendencies, borderline personality disorders, preschool children with less developed verbal communication, children with autistic spectrum disorders, and clients with mental handicaps. ‘Now I've been doing early care for children with autism for half a year. With that early care, because it covers the 0–7 age category, I think the physical contact, the eye contact, and the person being close are irreplaceable. That doesn't work online’ *(focus group – psychotherapy)*.

Distant care appeared to be more successful with existing clients. The practitioners found it harder with new contacts. ‘This was a bigger problem with new people, where it took me much longer to engage them or establish a relationship, to have them open up’ *(clinical psychologist, 49 years old)*. Some practitioners did not even take on new clients in the pandemic situation out of principle or could not imagine engaging them in distant contact. ‘It's definitely important that the first contact is face to face’ *(clinical psychologist, 30 years old)*. Organisations, too, showed an initially rather reserved and reluctant attitude to first‐contact clients. It was often a matter of practitioners' resolve as to how to address the issue. ‘We decided we wouldn't do new contacts, but I broke that rule several times. It was better than I had thought. In one case, I do feel that the first session with the client went extremely well, and we struck up a rapport, and the client is still in contact’ *(counselling psychologist, 56 years old)*.

### Experience changes attitudes and approach

The most positive aspect of our study is the finding that experience changes the attitude towards a problem. Most of the respondents had positive experiences with distant psychological care after the initial experimenting and uncertainty phase. ‘At the beginning, we actually faced a barrier in that the therapist just couldn't imagine providing care on a long‐distance basis. But we overcame this relatively quickly; it was a matter of two weeks or so’ *(focus group – clinical psychology)*.

However, the practitioners who tend to view the distant form of psychological care in favourable terms can be divided into two groups. The first and larger one consists of those individuals who use distant approaches while continuing to consider them an emergency measure only and not being able to imagine them in the future as a significant standard or alternative form of care: ‘To me, it's good as an alternative, but it's more of an emergency option’ *(clinical psychologist, 45 years old)*. The second and smaller group also finds the online modality a valuable alternative for the future. While being aware of certain limitations of such approaches, the representatives of this group also see their benefits and the potential for further exploration: ‘There's definitely nothing to worry about with that distant care. It's just progress. Moreover, like when email came in, it did not make the regular post extinct. When telephones came in, personal contacts did not vanish. The same thing will happen with videoconferencing now. It's another option we can use in certain situations. It has its pros. It has its cons. Among a certain group of patients, it may also be beneficial in certain situations’ *(clinical psychologist, 30 years old)*.

## DISCUSSION AND CONCLUSION

Recent evidence provides an overview of general broadly defined barriers to online services on the part of the providers (Dijksman et al., 2017; Gilmore & Ward‐Ciesielski, 2019; Schuster et al., 2018). A scoping review of mental‐health tele practice during COVID‐19 likewise identified resource constraints and concerns about therapeutic rapport as persistent obstacles (McBeath et al., 2020). A more recent scoping review of 81 studies across mental‐health systems likewise highlights resource constraints, regulatory complexity, and perceptions of digital care as ‘impersonal’ as persistent implementation hurdles (Berardi et al., 2024).

In line with recent research (Doran & Lawson, 2021; Gentry et al., 2021; Li et al., 2021; Machluf et al., 2021), we can conclude that following the initial phase of experimenting and uncertainty, most of the providers have had a positive experience with distant (online) care. There seems to be a trend of providers falling into those who consider online services an emergency solution only and those who see them as a promising alternative to face‐to‐face services in the future. Comparable trajectories have been documented in qualitative studies from the United Kingdom and the United States, where clinicians reported a rapid shift from provisional to routine tele practice once initial technical hurdles were overcome (McBeath et al., 2020; Pierce et al., 2021).

Online psychological assessment turned out to be a domain of its own, as its practical application depends heavily on the availability of online methods for specific areas of psychology, licensors, standardisation of methods for online use, and their affordability. Best practice guidelines that have been in place for more than a decade (Luxton et al., 2014) stress that psychometric equivalence between in‐person and remote administrations cannot be taken for granted, so clinicians must adapt their procedures to telehealth‐specific factors. When these standards are applied with adequate preparation and technology, remote assessments can attain reliability and validity comparable to traditional formats while simultaneously widening access to specialist and culturally matched services. Nevertheless, some of the participating experts still rejected using some methods online altogether. Referring to specific professional approaches as ‘impossible’, ‘unimaginable’, or ‘applicable purely exceptionally or in cases of emergency’ may often be hasty and limiting, especially when participants have never tried them for themselves. Such attitudes may reflect needless mental blocks stemming from lacking information and experience. However, further expert discussion is required to distinguish what a stereotype is and what an objective barrier is to providing specific psychological care online.

Our findings support the broader theory of the acceptance of new technology (Momani, 2020), which proposes, inter alia, that the acceptance of new procedures and technological approaches requires especially the presence of positive norms in society, the availability of a sufficient quantity of relevant information and procedures (guidelines), and the allowing of enough time for people to acquire positive experience of the new technology (in our case, especially the interaction through videoconferencing). Conversely, the absence of the circumstances outlined above may strengthen the role of prejudice and rigid decision‐making relative to the acceptance of the new technology. Practice showed that if a professional association issuing recommendations was represented by professionals with hands‐on experience with online care, their position tended to be encouraging. At the same time, where they had not tried this method of work for themselves, their recommendations were somewhat restrictive and limiting. This interpretation accords with findings from an earlier review synthesis demonstrating that, in spite of hesitancy among psychologists, even those with little experience in video therapy adapt their communication style and adjust to the technology in a relatively short period of time (Simpson & Reid, 2014).

We suggest that any efficient provision of high‐quality psychological support on a distant basis (online) requires an appropriate and supportive institutional framework, including technical resources, legislative background, and the dissemination of information. It is, therefore, essential that such conditions are quickly and flexibly achieved at all levels of governance (national authorities, local governments, and professional associations) and, importantly, that such steps are taken in response to the needs and input of those who provide such services (a bottom‐up approach). Previous research findings mentioned in the introduction also suggest the importance of external factors (Gentry et al., 2021; Gilmore & Ward‐Ciesielski, 2019; Muir et al., 2020; Pierce et al., 2021; Yoon et al., 2021) for the successful implementation of online services.

As experience progressed, many providers moved from being cautious experimenters to regular practitioners. Technical difficulties and negative stereotypical attitudes were receding. Many respondents were trying to find ways to continue providing their services for their client's benefit. The level of clear‐cut and dismissive opinions about contraindicated groups of clients decreased significantly as positive experiences with the provision of distant psychological care to these generally challenging clients grew. The descriptions of true contraindications were thus becoming more consistent with reality and the professional literature. For example, Stoll et al. (2020) in their narrative review note that online psychological care is particularly suitable for clients in rural or otherwise underserved areas, for individuals who are housebound or have limited mobility, and more generally for patients with mild to moderate symptoms; it can even serve as an interim option in acute crises when immediate face‐to‐face help is unavailable. Its appropriateness, however, is debatable for people with severe mental disorders, highly dysfunctional presentations, elevated risk of harm to self or others, or cognitive/technological limitations that make effective use of digital platforms difficult.

This study has three main limitations that should be borne in mind when interpreting its results. First, the sample comprised psychologists who volunteered to discuss their experiences of distance care; their willingness to participate may indicate a greater interest in, or stronger views about, online practice than is typical in the wider professional community, which could skew the balance of perceived barriers and benefits. Second, although the interviews were conducted soon after the major COVID‐19 restrictions had eased, they still relied on retrospective accounts of a rapidly evolving situation; some details may therefore be coloured by recall bias or by subsequent adaptations that were already under way when the interviews took place. Third, several findings are likely to be nation‐specific, reflecting the organisation, reimbursement rules, and licensing requirements of the Czech mental health system; while the procedural barriers we describe have parallels elsewhere, their precise configuration may limit direct transferability to jurisdictions with different regulatory and funding arrangements. Despite these constraints, the study offers an empirically grounded map of obstacles encountered during the swift shift to online psychosocial care and points to practice recommendations that can inform further comparative work.

Consistent with the principles of reflexive thematic analysis, we offer several practice recommendations for each theme generated.

Adaptational, logistic, and technical barriers: To meet practitioners' needs, comprehensive digital adaptation training programmes should be set up, accompanied by dedicated funding that allows nonprofit and public providers to acquire the necessary hardware and software. Services ought to prepare contingency plans for technical failures—such as an immediate switch to telephone contact—and deepen collaboration with IT specialists to streamline logistics. Equally important is reinforcing work–life boundaries, privacy protocols, and training aimed at preventing online fatigue, while investing in the development and licensing of validated online assessment instruments.

Barriers concerning therapeutic contact and embodiment: Clinicians require specific training in online nonverbal communication strategies and access to tools that enhance audiovisual clarity. Hybrid models combining in‐person and remote work can harness the advantages of each modality, provided client feedback on the online experience is gathered systematically. Practitioners should also be trained to manage emergencies in digital settings, supported by ongoing research into methods for fostering authenticity and rapport in virtual sessions. Finally, raising awareness of both the benefits and the limits of distance care will help embed it as a credible complement to traditional psychotherapy.

Ethical and legal barriers: Clear, unified guidance on the ethics and legalities of online therapy needs to be promoted, alongside investment in encrypted, user‐friendly platforms. Practitioners must be educated in digital‐consent procedures and encouraged to conduct regular privacy audits. Because telehealth legislation is fluid, services should monitor and adapt to emerging regulatory requirements, while advocating for reimbursement policies that put online interventions on an equal footing with face‐to‐face care.

Stereotypes versus experience: Negative preconceptions about the efficacy of digital care can be countered by disseminating documented success stories, encouraging open peer discussion, and presenting evidence‐based data on adaptability. Targeted training should equip clinicians to modify online care for client groups traditionally deemed unsuitable, thereby broadening access without compromising quality.

Briefly summarised, the logistical barriers are associated with poor management and insufficient political, legislative, and financial support. The ethical and legal barriers must be taken seriously by key players in the system. The question of embodiment, which means dealing with new therapeutic cues and compensating for the absence of the usual cues, is an extensive and discussed topic that follow‐up research should explore thoroughly. The barriers concerning biases and stereotypes, i.e., limitations that the providers seem to create for themselves and are products of their attitudes and beliefs rather than being logistically, methodologically or professionally insurmountable problems, can be solved by specific training and sharing experiences among professionals.

## AUTHOR CONTRIBUTIONS

**Miroslav Charvát:** Methodology; investigation; validation; formal analysis; visualization; writing – original draft. **Leona Jochmannová:** Conceptualization; funding acquisition; project administration; supervision; investigation; writing – review and editing. **Petra Zia Sluková:** Investigation; writing – original draft. **Lucie Viktorová:** Data curation; resources; investigation.

## CONFLICT OF INTEREST STATEMENT

The authors report that there are no competing interests to declare.

## Supporting information


Appendix S1.

## Data Availability

The anonymised transcripts of the interviews and focus groups used in the reflective thematic analysis that support the findings of this study are available solely in the Czech language. They are available at the request of reviewers from the corresponding author. These transcripts are not publicly released due to participant privacy concerns and ethical or legal restrictions. Reviewers may contact the authors for further details regarding the methodology and analysis.
