# Supplementary material for: Transition to online psychological support – Barriers, stereotypes and challenges from the perspective of service providers
Source: Psychol Psychother. 2025 Aug 27;99(1):113–28. doi: 10.1111/papt.70010 (PMC12905525; doi:10.1111/papt.70010)
Supplement: Supplementary file 1 — Appendix S1. [file PAPT-99-113-s001.docx]

# Interview Guide for the Project:

Options for Online Psychosocial Care in Situations Requiring Limited Direct Social Contact

## The interview is semi-structured; the guide, therefore, serves as an orientation framework and may be adapted during the conversation. The questions are exemplary and need not be asked in the exact wording or order provided. It is unnecessary to ask every participant every question; rather, explore in depth those areas in which the particular professional has experience.

## Informed consent of the participant

Informed consent must be obtained after a presentation of the aims of the study, data‑handling procedures, participant rights, etc.

## Header

- Sex (gender):
- Age:
- Field/profession and general employment:
- Years of practice:
- Brief context (setting) of the interview:

## Introductory section

- How would you evaluate distance care for clients in general?
- What is the main thing you take away from this experience?
- Could you summarise what distance care for clients means to you and how you view it?
- In your opinion, what are the differences between face‑to‑face work and distance work?
- (Let participants freely summarise their experience first. This should be a less structured part of the interview before we focus their attention on the areas that interest us.)

## Extent of experience with online care (not only during the COVID‑19 restrictions)

- How many remote contacts with clients have you had in total?
- What types of contacts or interventions were they?
- Counselling or crisis intervention?
- Therapy or psychotherapy?
- Diagnosis or assessment?
- Other – which? (e.g., group work, work with families, couples…)
- What worked well for you? What would you do the same way again? (ideally with concrete examples)

## Main challenges and problems with types of intervention

- Conversely, what would you do differently within the interventions and contracts mentioned?
- What interventions or contracts would you not attempt, and why?
- What did you not do? Why did you not do it? Why was it not possible? What would you need to do it?
- What troubled you most during remote contact with clients? What did you dwell on? (ideally with concrete examples)
- Where do you see (if any) the most significant difficulties in psychodiagnostics? (e.g., unusable methods, stimulus material, loss of validity…)
- Where do you see (if any) the most significant difficulties in psychotherapy? (e.g., missing non‑verbal cues, different experience of the therapeutic relationship…)
- Did remote contact with clients differ from the methods you usually use?
- Can you think of any ethical limitations or problems? (e.g., protection and security of privacy and confidentiality)

## Types of clients and their reactions

- How did clients themselves react to this change or situation?
- What concerns, feelings and attitudes towards distance care did they express?
- With what type of clients did you work successfully at a distance, and with which not?
- Diagnostic groups, children vs adults, older vs younger, individuals vs groups…
- Did you work only with existing clients, or did you also try entirely new clients?
- Do you see any possible contraindications? If so, for which client groups?
- Why would you not work with these client groups?
- Conversely, do you believe some clients can profit from such distance contact?

## Technical aspects

- What technical equipment did you use in distance care? (personal or work)
- What hardware and software, or which applications, proved helpful?
- For which functions did you particularly like them?
- What is the minimum technical set‑up required for distance psychosocial care?
- What would be the optimal equipment? (for instance, concerning assessment)

## Personal evaluation of the experience

- What was it like for you to work with clients remotely?
- How did you experience this rather unusual situation?
- What did you miss the most in distance work compared with what you are typically used to?
- What would help you feel more stable or safer in such a professional contact?
- What measures would you recommend regarding mental health and protecting professionals' private lives?
- How would you comment on working from home in this context (the so‑called home office)? (advantages, disadvantages, pitfalls)

## Final recommendations and evaluation – recap

- What would you chiefly recommend to others when using distance forms of contact?
- What should professionals not do, or do, in distance care, and how?
- What form of methodological support would you welcome most concerning this issue?
- What should not be missing from a planned guideline for distance care?

Is there anything else you would like to mention that I haven't asked about but that you consider essential? … Thank you for the interview.
